# Establishing multiple cost-effectiveness thresholds in Oman: a stakeholder-driven approach

**DOI:** 10.1186/s13561-026-00817-w

**Published:** 2026-07-15

**Authors:** Said Wani, Hilal Alsabti, Said Allamki, Ahmed Almandhari, Suleiman Alhajji, Ibrahim Alrashdi, Raid Alsabri, Adnan Alqassabi, Ahmed Alzaabi, Aisha Almaashani, Alia Alshuaili, Anisa Rasool, Asiya Alkindi, Bushra Salman, Faiza Alzadjali, Khalid Alrahbi, Khulood Almutawa, Manal Alansari, Mardheya Alkharusi, Nadiya Albulushi, Rahma Alghadani, Rana Aljaber, Safiya Almuaini, Said Alkindi, Salim Alzawamri, Sara Albalushi, Suha Allawati, Zuhair Alsalti, Hassan Albalushi, Ahmad Nader Fasseeh, Zoltan Kalo

**Affiliations:** 1 Center for Health Technology Assessment, Semmelweis University, Ulloi ut 25, Budapest, 1085 Hungary; 2 Center for Pharmacology and Drug Research & Development, Semmelweis University, Budapest, Hungary; 3 Ministry of Health, Muscat, Oman; 4 Royal Hospital, Ministry of Health, Muscat, Oman; 5 Pharmacy Department, Ministry of Health, Sohar, Oman; 6 Sultan Qaboos Hospital, Ministry of Health, Salalah, Oman; 7 Pharmacy Department, National Hematology and Bone Marrow Transplant Center, University Medical City, Seeb, Oman; 8 Medical Devices Control Department, Drug Safety Center, Ministry of Health, Muscat, Oman; 9 Pharmacy Department, Sumail Hospital, Ministry of Health, Sumail, Oman; 10 Khoula Hospital, Ministry of Health, Muscat, Oman; 11 Clinical Pharmacy, Al Nahdha Hospital, Khoula Hospital, Ministry of Health, Muscat, Oman; 12 Pharmaceutical Care Department, Ministry of Health, Muscat, Oman; 13 Ministry of Health, Rustaq, Oman; 14 Pharmacy Support Services, Sultan Qaboos Comprehensive Cancer Care and Research Centre , University Medical City, Seeb, Oman; 15 Khoula Hospital, Ministry of Health, Muscat, Oman; 16 Ministry of Health, Salalah, Oman; 17 Department, Ibri Hospital, Ministry of Health, Ibri, Oman; 18 Syreon Middle East, Alexandria, Egypt; 19 Faculty of Pharmacy, Alexandria University, Alexandria, Egypt; 20 Syreon Research Institute, Budapest, Hungary

**Keywords:** Cost-effectiveness threshold, CET, Oman, Health technology assessment, Economic evaluation, Multiple thresholds, Cost-effectiveness threshold multiplier

## Abstract

**Background:**

The rising cost of new health technologies and limited healthcare budgets challenge health systems worldwide, including Oman. Local value judgments are crucial for informed decision-making. This study aims to establish a cost-effectiveness thresholds (CETs) framework for Oman, aligned with the country’s economic status and healthcare priorities.

**Methods:**

The key findings from a recent literature review on global CETs and their applications were shared and discussed during a multi-stakeholder workshop in March 2024. Presenting these findings has provided participants a broad understanding of global CET practices and highlighted the use of multiple thresholds across different settings. Afterwards, a survey was distributed, asking participants to anonymously vote on six key concepts: the basis for the threshold, the baseline threshold multiplier, the use of multiple thresholds, the criteria for multiple thresholds, the number of multipliers, and which criterion should receive the 3X multiplier. The results from this voting guided the design and final agreement on the threshold values.

**Results:**

Twenty respondents supported adopting multiple CET values based on cost per quality-adjusted life-year (QALY), linked to Oman’s economic status. A baseline CET equal to 1x Gross Domestic Product (GDP) per capita was suggested. Participants recommended three different threshold multipliers to reflect policy priorities. A continuous multiplier (1–3x GDP), based on incremental QALY gains, was used to favor technologies with greater health gains. Additionally, a fixed multiplier of 2 was chosen for both orphan drugs and treatments for priority diseases. The resulting CET values reflect technical and expert consensus and do not include patient or public perspectives.

**Conclusions:**

In Oman, the incremental cost per QALY gained should not exceed 1x GDP per capita for new health technologies that offer minimal health gains in common diseases. However, thresholds might increase to 12x GDP per capita for curative treatments in rare priority disease areas. It is recommended that CET values be reviewed and updated within three years, based on initial implementation experiences.

**Supplementary Information:**

The online version contains supplementary material available at https://doi.org/10.1186/s13561-026-00817-w.

## Background

Over the past few decades, healthcare systems worldwide have been transformed by the rapid pace of medical innovation. New diagnostics, medical devices, and treatments, especially high-cost gene therapies and personalized medicines, have significantly improved health outcomes but also put unprecedented pressure on healthcare budgets [1–4]. Even in high-income countries like Oman, maintaining equitable access to these emerging technologies within limited financial resources is becoming increasingly challenging. Health policymakers worldwide, including those in Oman, face difficult decisions about which technologies to prioritize. In reality, no healthcare system can afford to reimburse all available health technologies on the market; therefore, decision-makers must make trade-offs to allocate resources efficiently.

Despite these challenges, Oman has achieved remarkable progress in healthcare development since the 1970s, significantly improving population health indicators through continued government investments and strong public health governance [4, 5]. In line with Oman Vision 2040, decision-makers aim to elevate the health system to international standards, guiding the country through a significant phase of healthcare transformation [6, 7]. However, as Oman advances this transformation, its healthcare system faces increasing pressure from a growing burden of chronic non-communicable diseases such as cardiovascular diseases, cancer, diabetes mellitus, chronic respiratory diseases, hypertension, obesity, and hypercholesterolemia [2]. Cancer remains one of the most significant public health challenges, with profound implications for health systems, economies, and societies. The economic burden of cancer is expected to increase further due to several reasons, including population aging and rising treatment costs.

In response, Omani policymakers are working to maximize health benefits from the limited available resources. They recognized the importance of implementing health technology assessment (HTA) to strengthen the evidence base for reimbursemaent decisions of new technologies [8, 9]. Accordingly, Oman has taken its first structured step toward institutionalizing HTA by developing a national HTA roadmap to guide the systematic implementation of HTA processes and to build local capacity for evidence-based policy [8].

HTA systematically evaluates the clinical, economic, social, and ethical implications of health technologies to inform resource allocation decisions [10]. Economic evaluation is a core component of HTA, judging the value of health technologies by comparing their health outcomes and costs. The main outcome of these evaluations is the incremental cost-effectiveness ratio (ICER), which quantifies the extra cost per unit of health gained, typically expressed in quality-adjusted life-years (QALYs) or disability-adjusted life-years averted (DALYs) [11].

However, for ICERs to be meaningful, they must be interpreted against a benchmark that reflects the marginal health benefits displaced elsewhere in the system when additional resources are allocated. This benchmark is known as the cost-effectiveness threshold (CET) [11]. If the ICER of a new intervention falls below this threshold, the technology is deemed cost-effective and potentially eligible for reimbursement. Conversely, if the ICER exceeds the threshold, the intervention is considered poor value for money [11]. Since several economic evaluations have been recently conducted and published in Oman, establishing CETs is crucial for translating economic evaluation results into actionable reimbursement decisions [12–14].

Recognizing this need, the Ministry of Health (MoH) in Oman identified establishing CETs as one of the initial, foundational steps in its HTA implementation plan [8]. This study aims to develop a framework of CETs in Oman that aligns with the country’s economic status and healthcare priorities.

## Methods

The CETs in Oman were developed using a comprehensive approach based on international experiences from recent literature reviews, while tailored to the local healthcare system. The establishment of the CET involved presenting the findings of Fasseeh et al.’s global review of CETs frameworks and Kovács’ work to national experts during a multi-stakeholder workshop [15, 16]. This step ensured participants gained a comprehensive understanding of CETs and their practical applications. Then, a structured survey-based voting exercise was conducted with local experts to gather their input on key CET elements, leading to a strong framework tailored to Oman’s specific needs and context.

### Multistakeholder workshop

A multistakeholder workshop was held in Muscat on March 4, 2024, involving participants from various government agencies with backgrounds in health economic evaluation. The selection of participants was based on convenience sampling, ensuring inclusion of individuals from different institutions with knowledge of HTA and involvement in decision-making. A target sample size of 30 participants (a minimum of 20 respondents) was set, following the adaptation of similar survey methods used in other countries [15, 17]. Participants were affiliated with different departments within the MoH and the University Medical City. Participants affiliated with the University Medical City were healthcare providers. This brought a wide range of expertise and perspectives that are important for healthcare decision-making in Oman. Table S1 shows the affiliations of participants included in the workshop.

An international expert with extensive experience in health economics and health policies moderated the workshop. The CET workshop started with a presentation of the results from a recent literature review on how CET was established and modified in other countries [15]. The literature review provided participants with an understanding of the different aspects of the CET, allowing them to make well-informed decisions about the final CET values presented in the survey.

Key conclusions from the review provided initial considerations for the CET framework in Oman [15, 16]. The review examined both the threshold values and their basis. CETs were further categorized by type—explicit or implicit—and variations in their reporting were assessed, including whether thresholds were presented as single estimates or ranges. Additionally, countries using multiple CETs were analyzed with attention to threshold values, national context, year of adoption, and the justification for using multiple thresholds. This comprehensive analysis provided a broad perspective on global CET practices, highlighting the use of multiple thresholds in some settings.

Based on the review findings, most countries’ threshold values were linked to their economic status, specifically to their Gross Domestic Product (GDP) per capita [15]. Higher-income countries had higher absolute threshold values [15]. While a single threshold value was the dominant approach in several countries initially, more countries have adopted multiple thresholds in recent years [15]. These countries favor technologies with higher relative health gains, especially for more severe diseases, orphan drugs, and priority disease areas such as cancer [15]. Among countries with multiple thresholds in the review, multipliers ranged from 3 times to 15 times the standard threshold [18–20].

### Survey

The survey instrument was developed based on a synthesis of international literature on CETs during the workshop planning phase. The survey was then distributed electronically to all workshop participants via Mentimeter^®^ software, which enabled anonymous live voting. Mentimeter allowed participants to submit their responses confidentially, and the results were displayed in real time once the presenter confirmed that all votes had been cast. Participants voted on six concepts, including: the basis of the threshold, the baseline threshold multiplier, the presence of multiple thresholds, criteria for multiple thresholds, the number of multipliers, and the criterion for the 3X multiplier. Each concept was presented as a question with multiple choices (see Table 1). The most voted option was used to define the consensus response, except for the baseline multiplier, where the median value was used to minimize outlier effects. Multiplier selection was guided by internationally recognized multipliers, preventing the threshold from being set too low or too high, with a maximum multiplier of 3X for only one criterion. Consent to participate and publish data was obtained from all participants before voting, as the initial step, using Mentimeter^®^ software. Participants were also allowed to propose additional response options when considered appropriate.

**Table 1:** Multi-stakeholder survey questions, voting options, and responses

1- What should be the basis for the cost-effectiveness threshold (CET)? (Single Response)		
Options	**Votes**	%
No need to conclude on cost-effectiveness thresholds, as value judgement should focus on clinical benefits only	0	0%
Threshold value should be linked to the GDP per capita	**20**	**100%**
Threshold value should be calculated by a different methodology	0	0%
2- What should be the baseline threshold compared to the GDP per capita? (Single Response)		
Options	**Votes**	%
0.50 GDP/capita	1	5%
0.75 GDP/capita	0	0%
1.00 GDP/capita	**11**	**55%**
1.25 GDP/capita	7	35%
1.50 GDP/capita	1	5%
1.75 GDP/capita	0	0%
2.00 GDP/capita	0	0%
3- Is there is a need for multiple thresholds? If yes, what should multiple thresholds be based on? (Multiple Response)		
No need for multiple threshold value, cost-effectiveness threshold should be same for all patients & technologies	0	0%
Patients with a rare disease deserve higher threshold value	13	65%
Patients with more severe diseases deserve higher threshold value	4	20%
Technologies with major improvement in health deserve higher threshold value than those with minor	10	50%
Patients in a selected priority disease (e.g. cancer) deserve higher threshold value	15	75%
4- How many multipliers would you like to define your CET? (Single Response)		
1	0	0%
2	4	20%
3	**16**	**80%**
5- Which criteria deserves a 3X multiplier? (Single Response)		
Relative health gain (IRQG)	**8**	**40%**
Rarity	5	25%
Priority diseases	4	20%
None	3	15%

This table shows the detailed results of the structured survey conducted during the national multi-stakeholder workshop held in Muscat in March 2024. The survey aimed to gather expert opinions on the design and specific multipliers for the CET framework in Oman. Participants voted anonymously using the Mentimeter^®^ platform on six key areas: the methodological basis of the CET, the baseline threshold value relative to GDP per capita, the need for multiple thresholds, the criteria justifying multiple thresholds, the number of multipliers to define the CET, and the criterion eligible for the maximum (3×) multiplier. The table includes the survey questions, response options, the number of votes for each, and the percentage of total votes. These results serve as the basis for establishing Oman’s national CET framework, considering economic status, priority disease areas, and factors such as disease rarity and relative health gains

Bold values indicate the questionnaire responses selected by participants and highlighted in the presentation of the survey results

The survey was designed to follow a flowchart-like structure, where subsequent questions were presented based on participants’ previous answers. This approach aimed to ensure the questions were relevant and the voting process flowed smoothly.

### Data analysis

Responses to each survey question were exported from the Mentimeter platform, compiled, and summarized using descriptive statistics. As noted earlier, the option that received the highest number of votes was adopted in the framework, except for the baseline CET multiplier, where the median value was used to reduce the impact of outliers.

## Results

### Survey results

Twenty participants attended the workshop and completed the survey. The voting results showed that all participants (100%) selected that the threshold value should be linked to Oman’s economic status, as represented by GDP per capita. For the baseline CET value, the median choice was 1×GDP per capita.

All respondents (100%) preferred using multiple thresholds. These thresholds were based on multipliers of the baseline threshold. The great majority of respondents (75%) preferred the multiple thresholds to be based on priority disease areas, 65% on disease rarity, 50% on relative health gain, and 20% on disease severity. Such a question allowed multiple responses, where participants could choose more than one criterion. Following this question, participants voted on the number of multipliers to be used in defining the CET. According to the survey results, 80% of respondents selected a maximum of 3 multipliers (corresponding to 3 criteria), while the remaining 20% chose a maximum of 2 multipliers. Therefore, consensus was reached on using three multipliers.

To align with internationally observed multipliers and considering Oman’s high GDP per capita, a maximum multiplier of 15 times the GDP per capita should not be exceeded, consistent with global standards [15]. Therefore, only one criterion was allowed a 3x multiplier, while the other two criteria were limited to 2x for each. This results in a combined value of 12 × (3x2 × 2), which stays within the 15x limit, whereas a combination of 3 × 3 × 2 would exceed the maximum multiplier, reaching a total of 18 multipliers.

To determine the criterion with the 3X multiplier, the highest three criteria preferred by respondents to have multiple thresholds (Q3) were added as options, and the rest were removed (disease severity was removed as it was not among the top three). Regarding the votes for the criterion deserving the 3X multiplier, relative health gain received the highest number of votes (40%) and therefore received the 3X multiplier. Rarity was determined according to the FDA’s orphan designation of the drug [21], while priority diseases were identified based on the Ministry of Health’s annual health report and national health survey [22, 23]. Some cancer types are included in the Ministry of Health’s report; however, the report contains other diseases. Table 1 presents the final survey questions and voting options, along with the number of participants who voted for each option.

### Accepted cost-effectiveness thresholds

Based on the survey results, the MoH drew the following conclusions regarding the cost-effectiveness thresholds.

The ICER should be compared to the appropriate threshold value, which is based on cost per QALYs and linked to Oman’s economic status. The baseline CET equals the most recent 1x GDP per capita expressed in Omani Rial (OMR) and is sourced from the Central Bank of Oman (CBO) or the National Centre for Statistical Information (NCSI). It is important to note that converting GDP per capita from international sources (e.g., the World Bank, the International Monetary Fund, or the World Health Organization) and currencies (e.g., USD PPP) is not considered a suitable reference value.

The CET should align with healthcare priorities, acknowledging that the willingness to pay for a QALY gain is higher for orphan medicines, medications for other priority diseases (for example, cancer), and technologies providing significant relative health benefits.

The CET is thus calculated using the following formula:

$$\begin{aligned}CET\:\left(local\:currency\right)&=1\times\:GDP/\\&Capita\times\:Multiplier\end{aligned}$$

The threshold multiplier for the high Incremental Relative Health Gain (IRQG), which refers to the proportion of additional health benefit relative to the total expected QALYs of the new technology, should be calculated using the same comparator, time horizon, and modelling assumptions as in the baseline economic evaluation. This information is clearly outlined in the Oman HTA guidelines [24]. The following formula explains the IRQG: 

$$\begin{aligned}IRQG=(\mathrm{Q}\mathrm{A}\mathrm{L}\mathrm{Y}\mathrm{n}\mathrm{e}\mathrm{w}&\:\mathrm{t}\mathrm{e}\mathrm{c}\mathrm{h}\mathrm{n}\mathrm{o}\mathrm{l}\mathrm{o}\mathrm{g}\mathrm{y}\:-\:\mathrm{Q}\mathrm{A}\mathrm{L}\mathrm{Y}\mathrm{c}\mathrm{o}\mathrm{m}\mathrm{p}\mathrm{a}\mathrm{r}\mathrm{a}\mathrm{t}\mathrm{o}\mathrm{r}\:\mathrm{t}\mathrm{e}\mathrm{c}\mathrm{h}\mathrm{n}\mathrm{o}\mathrm{l}\mathrm{o}\mathrm{g}\mathrm{y})\:\\&/\:\mathrm{Q}\mathrm{A}\mathrm{L}\mathrm{Y}\mathrm{n}\mathrm{e}\mathrm{w}\:\mathrm{t}\mathrm{e}\mathrm{c}\mathrm{h}\mathrm{n}\mathrm{o}\mathrm{l}\mathrm{o}\mathrm{g}\mathrm{y}\end{aligned}$$ [16]

**Fig. 1:**
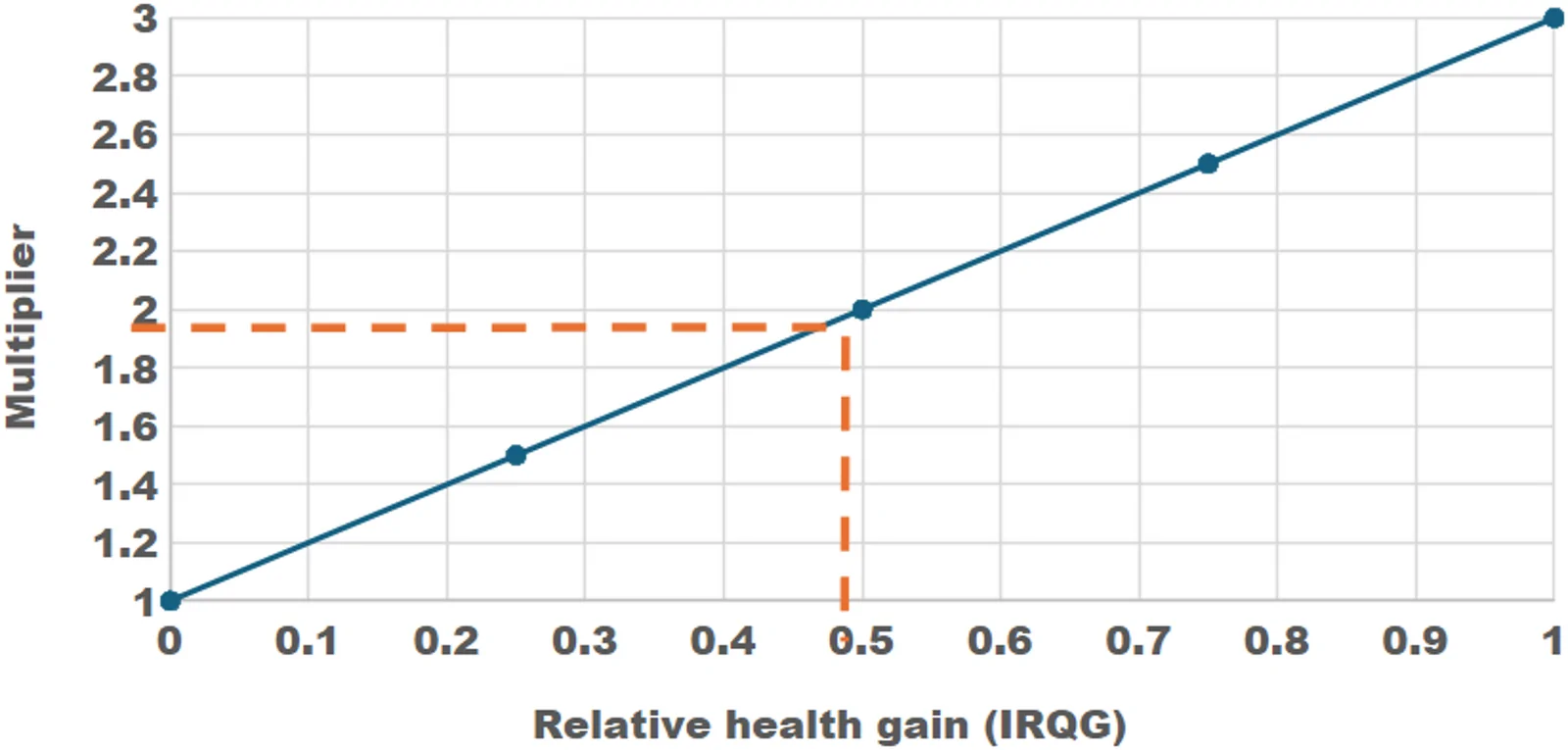
IRQG multiplier

Using QALY of new technology as the denominator shows how the incremental benefit adds to the overall health outcome, allowing comparison between technologies with similar absolute gains but different relative improvements [16]. The threshold multiplier for relative health gain is a continuous variable that ranges from 1 to 3. For instance, when the IRQG is 0.1, the CET is multiplied by 1.2 (1 + (0.1 × 2)), following a linear scale. For an IRQG of 0.25, the CET is multiplied by 1.5 (1 + (0.25 × 2)), and so on. A multiplier of 1 reflects no relative health gain, while a multiplier of 3 represents the highest relative health gain. Figure 1 illustrates the continuous approach applied to IRQG. A fixed multiplier of 2 is used for both orphan drugs and medicines treating priority diseases (such as cancer treatments). A link for CET calculation is found in the supplementary material.

In HTA submissions, the relevant CET should be displayed in a table format, including the most up-to-date GDP per capita in OMR and the baseline threshold in OMR. The table should then specify the multiplier for IRQG, reflecting the relative health gain associated with the intervention. Additionally, the multiplier for disease rarity should be reported when the condition under assessment qualifies as a rare disease, and the multiplier for disease priority should be indicated when the intervention targets a nationally prioritized disease area, such as cancer. An aggregate threshold multiplier should subsequently be calculated by combining the effects of the IRQG, disease rarity, and disease priority multipliers. Finally, the resulting CET in OMR should be presented, representing the final threshold value obtained by multiplying the baseline threshold by the aggregate multiplier.

## Discussion

Implementing HTA is crucial for helping stakeholders make evidence-based decisions. The cost-effectiveness threshold represents the maximum monetary value that a health system is willing to pay for a single unit of health benefit, such as a QALY, gained from a health technology. Our study established a CET tailored to Oman’s specific economic and healthcare system to inform the reimbursement decisions in the country.

Empirical estimates may establish CET thresholds if previous economic evaluations of health technologies can be linked with policy decisions [11]. This approach was not feasible in Oman because there were no prior economic evaluations. Additionally, if experience-based thresholds are not regularly updated in light of recent policy decisions, CETs may lose connection with opportunity costs on the supply side.

The threshold values can theoretically be established based on contingent valuation studies to explore the Willingness to Pay (WTP) for a one QALY gain [11]. Due to the limited number of Omani experts with a proper understanding of CETs and their implications, a large-scale WTP survey was not considered an appropriate approach to establish CETs in Oman.

Due to the limitations discussed above, there is a worldwide shift toward customizing CETs to reflect each country’s economic status, healthcare priorities, and societal values. Several studies have shown the adoption of GDP-based thresholds with different ranges. A comprehensive review by Leech et al. indicated that 66% of public research on cost-effectiveness analysis from 2000 to 2015 established a connection between CET and GDP per capita [25]. The MoH in Oman decided to adopt the GDP-based CET approach, reflecting its economic status. Oman is the fourth country in the Middle East after Egypt, Saudi Arabia, and the United Arab Emirates (UAE) with explicit CETs [15, 17, 26].

Oman’s CET allows preferential consideration or the application of higher multipliers for orphan drugs targeting rare diseases and for priority disease areas such as cancer treatments. This approach is similar to CET frameworks in France, Italy, and Canada for cancer treatments as well as with frameworks in Thailand, France, the USA, and the United Kingdom for rare diseases and orphan drugs [15, 27–30].

Comparing the approach used in Oman with those in Egypt, the UAE, and Saudi Arabia shows both similarities and differences. Like Oman, the CETs of Egypt and the UAE are linked to GDP per capita, and the multiple thresholds were derived from localizing the findings of literature reviews through multistakeholder workshops [15, 17]. In contrast, Saudi Arabia’s approach uses evidence on the income elasticity of the value of health, with the CET estimated at SAR 50,000 to SAR 75,000 per QALY gained [26]. The Saudi approach relies on evidence from other high-income countries, which might not be fully applicable to Saudi Arabia’s mixed public and private healthcare system [26].

Implementing the CET framework in Oman is expected to bring about significant improvements in resource allocation, decision-making, transparency, accountability, equitable access to healthcare, and informed policy development. These outcomes are likely to lead to a more efficient, effective, sustainable, and equitable healthcare system in Oman.

Our study has certain limitations. Stakeholder perspectives were gathered from a single workshop, which may not fully represent the views of the broader population. Furthermore, the study did not involve any patient representatives in developing the CET. We acknowledge the importance of including patients and their representatives, but in Oman, such groups are still in their early stages. Additionally, no representatives from the Ministry of Finance were included. Although the GDP-based threshold in Oman is flexible and responsive to fluctuating oil revenues, providing a starting point for establishing cost-effectiveness thresholds, it has limitations. Specifically, GDP-based thresholds do not capture the true health opportunity cost and may not reflect the marginal productivity of healthcare spending. Consequently, such thresholds may overestimate the actual willingness-to-pay. In Oman, the absence of historical data connecting cost-effectiveness to funding decisions makes it difficult to link the threshold to opportunity costs; therefore, a GDP-based approach was used. It is recommended that CETs be reviewed based on initial experiences as part of the Oman Ministry of Health’s plan to update the HTA methodological guidelines within 3 years, ensuring that thresholds remain aligned with healthcare system priorities and broader macroeconomic conditions. Additionally, future revisions could incorporate the perspectives of other stakeholders, including patients, if patient groups become more established.

## Conclusion

The study established an explicit CET framework for Oman. The baseline threshold is linked to GDP per capita and is complemented by multipliers that consider priority disease areas, rarity, and health gains. This stakeholder-driven approach ensures that HTA in Oman is both economically sound and contextually relevant to the local context, supporting better healthcare decisions and reflecting diverse viewpoints from the healthcare sector.

## Supplementary Information

Supplementary Material 1.

## Data Availability

The original contributions of this study are included in the article. For further questions, please contact the corresponding author.

## Abbreviations

HTA: Health technology assessment
CET: Cost-effectiveness threshold
ICER: Incremental cost-effectiveness ratio
MoH: Ministry of health
GDP: Gross domestic product
QALYs: Quality-adjusted life-years
DALYs: Disability-adjusted life-years
CETs: Cost-effectiveness thresholds
IRQG: Incremental relative QALY gain
OMR: Omani rial
CBO: Central bank of oman
NCSI: National centre for statistical information
USD: United states dollar
PPP: Purchasing power parity
WTP: Willingness to Pay
UAE: United arab emirates
