# Supplementary material for: Establishing multiple cost-effectiveness thresholds in Oman: a stakeholder-driven approach
Source: Health Econ Rev. 2026 Jul 15;16:113. doi: 10.1186/s13561-026-00817-w (PMC13656365; doi:10.1186/s13561-026-00817-w)
Supplement: Supplementary file 1 [file 13561_2026_817_MOESM1_ESM.docx]

Establishing Multiple Cost-Effectiveness Thresholds in Oman: A Stakeholder-Driven Approach

Table S1 Workshop participants

| Experts | Affiliation | Responsibility |
| --- | --- | --- |
| 1 | Directorate General of Medical Supplies, Ministry of Health | Payer |
| 2 | Directorate General of Medical Supplies, Ministry of Health | Payer |
| 3 | Royal Hospital, Ministry of Health | Payer |
| 4 | Sohar Hospital, Ministry of Health | Healthcare provider |
| 5 | Directorate General of Medical Supplies, Ministry of Health | Procurement |
| 6 | Directorate General of Medical Supplies, Ministry of Health | Procurement |
| 7 | Directorate General of Medical Supplies, Ministry of Health | Quality |
| 8 | National Hematology and Bone Marrow Transplant Center, University Medical City | Healthcare provider |
| 9 | Director of the Medical Devices Control Department, Ministry of Health | Regulatory |
| 10 | Samail Hospital, Ministry of Health | Healthcare provider |
| 11 | Directorate General of Khoula Hospital, Ministry of Health | Healthcare provider |
| 12 | Directorate General of Medical Supplies, Ministry of Health | Payer |
| 13 | Al Nahdha Hospital, Ministry of Health | Healthcare provider |
| 14 | Directorate General of Medical Supplies, Ministry of Health | Pharmaceutical care, Regulator |
| 15 | Directorate General of South Albentina,Ministry of Health | Healthcare provider |
| 16 | Sultan Qaboos Comprehensive Cancer Care and Research Centre, University Medical City | Healthcare provider |
| 17 | Directorate General of Medical Supplies, Ministry of Health | pharmaceutical supply |
| 18 | Al Nahdha Hospital, Ministry of Health | Healthcare provider |
| 19 | Directorate General of Health Services, Ministry of Health | Regulatory |
| 20 | Director in the Directorate General of Planning, Ministry of Health | Planning |

Link for the CET Calculator in Oman: <https://syreon.me/cet_calculators/cet_oman.html>
